# Case of Congenital Hypertrophy of the Tongue, and Amputation

**Published:** 1858-07

**Authors:** C. Morrogh

**Affiliations:** New Brunswick, N. J.


### ARTICLE XIV.

*Case of Congenital Hypertrophy of the Tongue, and Amputation.* By C. Morrogh, M. D., of New Brunswick, N. J.

In May, 1857, a daughter of Mr. Joseph Mather, of Cranberry, aged seven years, was brought to me for advice, regarding a congenital enlargement of the tongue.

At birth the hypertrophy was moderate. but it had increased more or less rapidly till reaching its present dimensions, I found the tongue protruding two inches outside the jaws. It measured two inches across at the teeth, and was of a corresponding thickness. The papillæ of the protruding portion were enlarged, and the mucous membrane was thickened and indurated. On the under surface was a ragged hard ulcer, produced by the pressure of the teeth. These were pressed forward considerably out of their natural position. The horizontal rami of the inferior maxilla were curved downwards, so that when the molar teeth came in contact, a space of about one inch remained between the upper and lower incisors. By this provision of nature, the girl was enabled to masticate her food, and swallow without difficulty. Excepting for this grievous and unsightly deformity, the patient was a fine comely girl, and enjoyed excellent general health. She had been treated by astringent applications and internal medicines, but without any avail. I proposed amputation as the only remedy, to which the parents acceded, and, on the 12th of the month, the child was brought to New Brunswick for the purpose of undergoing the operation.

In 1829, Dr. Harris, of Philadelphia, endeavored to amputate in a similar case by ligature. He found that the circulation became quickly restored every time he strangulated the protruding portion by tightening the ligature. In this way the process became so severely painful and tedious, that he completed the operation with a catlin, cutting directly through the furrow made by the ligature. The arteries were tied, and the case progressed favorably. In 1835, another case offering itself, he amputated by a double flap incision, checking the hemorrhage by ligature, and forming a pointed well-shaped tongue. Thus did this eminent surgeon dispel those terrible phantoms of hemorrhage which had led practitioners into the barbarous method of removing portions of the tongue by strangulation.

I resolved to follow the last proceeding of Dr. Harris, with the exception of the ligatures, which I thought might be dispensed with, by using the needle and twisted suture.

On the day the patient was brought to town, I proceeded to operate, assisted by Drs. Taylor and Baldwin. The patient was rendered insensible by chloroform, and placed on her side upon a table, so as not to allow any blood to trickle through her fauces. A stout thread was then passed through the tip of the tongue, with which it was pulled forward, the frænum was then divided, and a strong pointed scissors applied, with one blade passed for some distance between the floor of the mouth and the under surface of the tongue, and the other on the dorsum. In this way a clean diagonal cut was made from the lateral edge to the raphe. At this stage of the proceeding, the hemorrhage was so free, I deemed it prudent to ligate the cut ranine artery, lest, after losing control of the organ, the sutures might fail in arresting the bleeding.

By a similar incision to the last on the other side, a V shaped portion was removed.

Without waiting to apply any more ligatures, two bead-headed needles were applied through the tongue, and the flaps approximated by the twisted suture. This measure immediatly arrested the hemorrhage.

In the evening the child detached the ligature by pulling at it. This led to a copious hemorrhage, which subsided and recurred several times. The child became feeble and feverish. The pulse 130, and jerking. After considerable trouble, the bleeding ceased altogether, and the child gradually improved.

On the third day the needles were removed. The wound had mostly healed by primary union, and on the sixth day, the patient went home well. Since that time, the end of the tongue has diminished to its natural thickness, and when at rest, it lays in its proper bed, with the tip behind the teeth. The curve of the lower jaw has much diminished and the appearance of the girl is very much improved. She can articulate plainly.

Should another case offer itself, my experience from this one would suggest the following plan of operation:

Pass each end of a silk thread through the lateral edges of the tongue, a little behind the position of the lower teeth. Let the ends and middle of the thread be drawn forwards together, so as to protrude the organ, and leave the centre free to be removed by a V shaped incision, having the apex backwards.

This is best done with a long sharp-pointed scissors, after first separating the tongue from the floor of the mouth carefully with a bistoury.

By this method of applying the suture, the tongue is still held under control after the anterior portion is separated.

By letting go the bite, or middle of the thread, and drawing on the ends, the flaps are immediately approximated, and should be secured by the sailor's reef knot. The tongue must then be again protruded, and quickly transfixed by a hare-lip needle, as near in the line of the ranine arteries as possible. A few twists of silk on the lower and upper surfaces of the tongue, will complete the suture, and effectually check the bleeding. The thread should not be twisted any tighter than is just necessary to effect this result, and bring the cut surfaces into contact.

In this manner the evil effects of ligatures may be dispensed with.—*N. J. Med. and Surg. Reporter.*

